# A global health opportunity: The potential of multiplexed diagnostics in low-resource settings

**Published:** 2011-12

**Authors:** Thor A. Wagner, Courtney A. Gravett, Michael G. Gravett, Craig E. Rubens

**Affiliations:** 1 Seattle Children’s, Seattle, Washington, USA; 2 Department of Pediatrics at University of Washington School of Medicine, Seattle, Washington, USA; 3 Global Alliance to Prevent Prematurity and Stillbirth, an initiative of Seattle Children’s, Seattle, Washington, USA; 4 Department of Obstetrics and Gynecology, University of Washington, Seattle, Washington, USA

Diagnostic assays typically quantify a single biomarker. There are theoretical benefits to assaying multiple biomarkers simultaneously, but the studies necessary to systematically identify and validate combinations of biomarker are usually large, complicated, and expensive. Not surprisingly, there has been little effort to develop multiplexed diagnostics for use in low-resource settings. However, emerging technology may soon make simultaneous testing for multiple biomarkers feasible in formats applicable to low-resource settings. The diagnosis of serious neonatal infections is a focus of this issue of the *Journal of Global Health*, and a good example of a clinical condition that has the potential to benefit substantially from multiplexed diagnostics.

Serious neonatal infections are responsible for nearly one million deaths per year (1). Early recognition of sepsis by a health care provider is critical for early initiation of treatment but is often hindered because newborns present with nonspecific signs and symptoms. Failure to rapidly and accurately diagnose serious neonatal infections can lead to delays in treatment and increased morbidity. Recognizing the potential to improve the diagnosis of serious neonatal infections, many researchers have evaluated biomarkers to more quickly and accurately diagnose sepsis in newborns. In this issue of the *Journal of Global Health,* we review the most promising new and emerging biomarkers for serious neonatal infections, and identify biomarkers with good diagnostic performance that seem to warrant evaluation in larger, future studies. We also looked at the few studies that examined the diagnostic potential of combination biomarkers, and found that select combinations of biomarkers showed promising performance.

There is good rationale for developing multiplexed biomarkers for the diagnosis of severe neonatal infections. Serious neonatal infection, also known as “neonatal sepsis”, is not a single disease, but represents a variety of infections, due to different pathogens, which lead to complex immunologic and physiologic responses, resulting in clinical syndromes with similar features. This is reflected by the fact that hundreds of individual biomarkers have been associated with “sepsis”. In this setting, focusing on a single biomarker from a single time point is potentially problematic. Using a panel of well-selected biomarkers that are not closely correlated and have different kinetics should improve the sensitivity, specificity, reproducibility, and the effective time-window of the diagnostic assay. For example, combining biomarkers that appear soon after infection, such as IL-6, with those that increase later, such as CRP, could improve the time range during which the test performs well (2). Alternatively, combining biomarkers from distinct pathways in the pathogenesis of sepsis, such as mediators of inflammation with mediators of coagulation may increase the specificity of a diagnostic test for severe neonatal infections. A panel of biomarkers may also provide information beyond predicting the likelihood of sepsis. Including biomarkers known to be associated with sepsis severity could provide important information for risk stratification of infected newborns, while using biomarkers associated with different types of pathogens may guide treatment decisions before culture results are available (3). Selecting biomarkers that are inversely correlated may help control for variability in specimen concentration and processing, which could cause correlated errors in biomarker measurement. Finally, combining established biomarkers is also attractive because there has already been extensive screening of these biomarkers, and combining promising biomarkers may provide a means to efficiently leverage diagnostic performance without the need for additional biomarker discovery effort.

**Figure:**
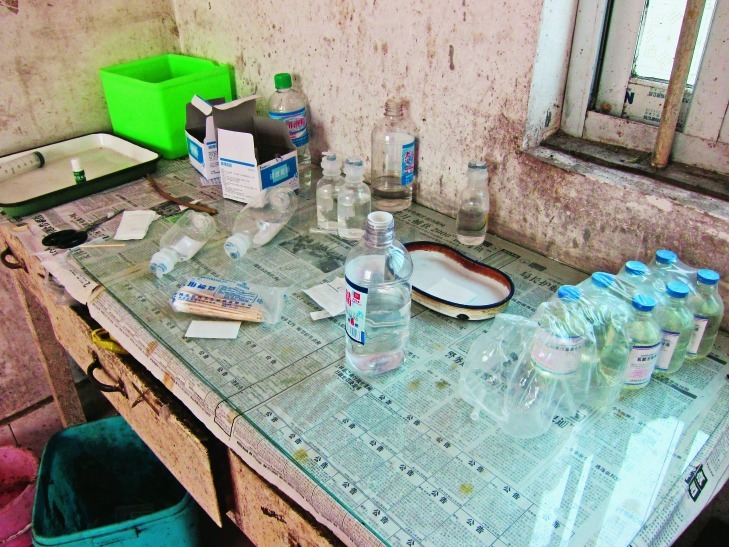
Photo: courtesy of Dr Kit Yee Chan, personal collection

Also supporting the concept of using a combination of biomarker is the failure for any single biomarker to demonstrate sufficient sensitivity and specificity to be adopted as a gold standard for the diagnosis of neonatal sepsis. The lack of a “gold standard” is a challenge to developing diagnostics for serious neonatal infections. Sepsis is best confirmed by blood or cerebral spinal fluid culture, but culture is neither 100% sensitive nor 100% specific. Cultures can be negative when the clinical picture is felt to be consistent with sepsis, and organisms that are potentially contaminants can also grow in culture. A review of neonatal sepsis studies found that cultures were positive in only 8% to 73% of neonates treated for sepsis (4). Relying on clinical findings is problematic because even in combination with laboratory values such as CRP, clinical findings can have low sensitivity (30-60%) and specificity (60-90%) (5). A well-designed multiplexed assay may have the potential to be both more sensitive and more specific than existing tests. This would be especially attractive for low-resource settings where the burden of neonatal sepsis is high and the infrastructure necessary for microbiologic culture is often severely limited.

**Figure 1:**
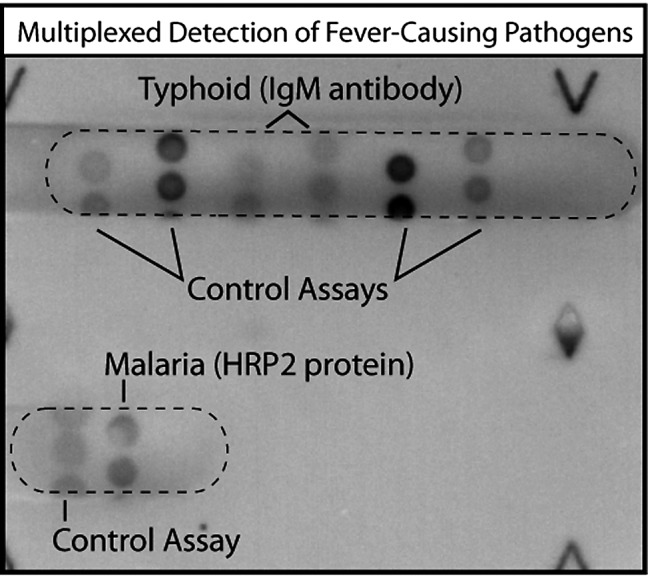
Multiplexed detection of both antigen and antibodies from a spiked blood sample. IgM specific to *Salmonella typhi* was detected in parallel with antigen specific to *Plasmodium falciparum* (malaria). Separate sample processing (specimen dilution and IgG removal) and two separate assay types were performed automatically on the same card. Dry reagents stored on the card were used to minimize user steps. This work was part of the DxBox, a project to perform sample-to-result identification of multiplexed fever-causing pathogens using a pneumatically actuated point-of-care device.

There have not yet been large scale efforts to develop multiplexed diagnostic tests for use in low-resource settings. However, emerging technology may soon make simultaneous testing for multiple biomarkers feasible even in low-resource settings. Recent technological improvements have fueled excitement for multiplexed diagnostic assays that can detect multiple pathogens or host markers in a single specimen (6). It is currently possible to build several test lines into a lateral flow strip, and newer technologies allow multiple independent assays to be included within a single lateral flow device (7-9). Furthermore, integrated microfluidic diagnostics have been successfully designed and engineered for use in low-resource settings. For example, see Figure 1 which shows multiplexed detection of both antigen and antibodies from a spiked blood sample. Specifically, *Salmonella typhi* IgM was detected in parallel with *Plasmodium falciparum* antigen. Both of these pathogens are common causes of fever in certain low-resource settings and bundling them together in a simple to use diagnostic should simplify and improve care in those regions.

Significant resources have been dedicated to research and development of related diagnostic technology, and it seems likely that sophisticated, multiplexed diagnostics will become increasing available in point-of-care formats. These newer diagnostics tend to utilize relatively small volumes of reagents, and by evaluating multiple biomarkers in a single device, the cost per biomarker evaluated should decrease significantly. Cost may be the most critical performance variable for diagnostic assays intended for use in low-resource settings. Most studies on novel biomarkers of sepsis evaluated the diagnostic performance of an assay without consideration of cost. A few investigators did estimate the cost per assay for the biomarker under evaluation; Ng et al measured blood serum IP-10 levels for US$ 6.55 (€ 4.9) per test using a flow cytometry bead assay (10), Cetinkaya et al evaluated serum levels of CRP or SAA for US$4 (€ 3) per test, and PCT levels for US$ 16 (€ 12) (11). Diagnostic tests ranging from US$ 4-16 (€ 3-12) may not represent a significant cost in resource-rich settings, but in many African countries, where the burden of neonatal sepsis is high, the average annual health care expenditure per capita is less than US$ 10 (€ 7.5) (12). Furthermore, the costs per assay reported above do not reflect all the costs associated with a laboratory-based assay. Most research studies are performed by skilled personnel in quality-controlled laboratories using sophisticated equipment that is also expensive to maintain. However, an ideal diagnostic test for neonatal sepsis would not rely on laboratory infrastructure, equipment, or personnel. Recent progress on developing two dimensional paper networks, allows automation of multi-step chemical processing and is being applied to antigen and IgM assays as part of the DxBox project (see **Figure 1**). Inexpensive technology offers great potential for multiplexed point-of-care diagnostic assays in resource limited settings. For example, a recent paper by Martinez et al describes an inexpensive paper cartridge used in microfluidic paper-based analytical devices (uPADS) that could be produced for less than $US 0.01 (for paper and patterning) (8). Diagnostic technology in this price range would make multiplexed diagnostic assays feasible even in low-resource settings.

**Figure:**
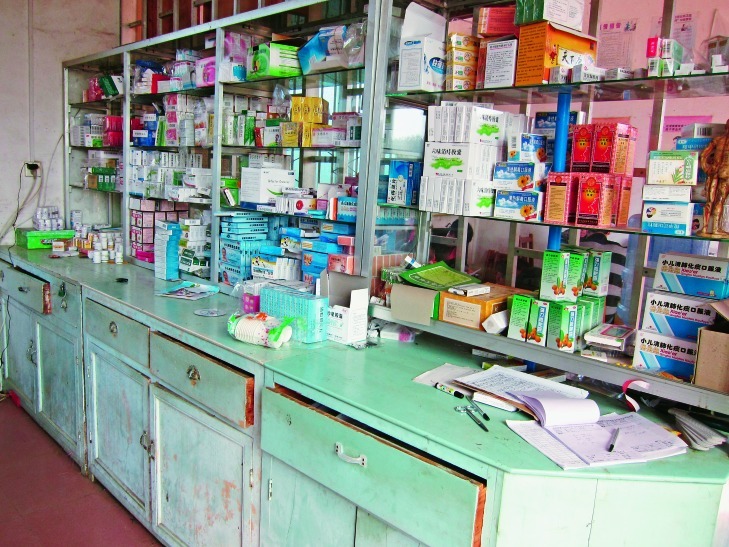
Photo: courtesy of Dr Kit Yee Chan, personal collection

There are many reasons to expect multiplexed diagnostics to outperform single biomarkers. Recent technological improvements are beginning to make the cost of multiplexed point-of-care diagnostics a possibility in low-resource settings. Rigorous evaluation of combined biomarkers requires large, complicated, and expensive studies, but when feasible, future diagnostic studies should be designed to evaluate multiple biomarkers. It is especially important to advocate for these types of studies in low-resource settings, where there is less commercial interest but significant potential clinical benefit to improved diagnostics. We encourage all future studies of diagnostics for severe neonatal infections in low-resource settings to be designed with sufficient scale and statistical input to rigorously evaluate combinatorial biomarkers.
